# Any role for respiratory allergens in IBS symptoms? From pathogenetic hypothesis to therapeutic implications

**DOI:** 10.3389/falgy.2026.1844274

**Published:** 2026-06-02

**Authors:** Carlo Maria Rossi, Marco Vincenzo Lenti, Antonio Di Sabatino

**Affiliations:** 1 First Department of Internal Medicine, Fondazione IRCCS Policlinico San Matteo, Pavia, Italy; 2 Department of Medicine and Medical Therapeutics, Università di Pavia, Pavia, Italy

**Keywords:** allergy, atopy, gut, therapy, variant

## Abstract

Irritable bowel syndrome is a common disorder associated with substantial impairment in quality of life and remains challenging to manage due to its heterogeneous and incompletely understood pathophysiology leading to high rates of treatment failure. Recent evidence supports the existence of distinct IBS endotypes, including an emerging atopic variant characterised by allergic comorbidities, type 2 inflammation, and mast cell activation and possibly response to mast cell-directed therapies. Mast cells appear to play a central role in symptom generation by modulating intestinal permeability, motility, and visceral sensitivity, and through close interactions with enteric nerves. Beyond established triggers such as stress and diet and food-related factors, growing attention has been directed towards the role of aeroallergens in influencing symptom variability. Epidemiological, clinical and translational data suggest a link between respiratory allergy and gastrointestinal symptoms. Identifying this phenotype may have important diagnostic and therapeutic implications, supporting the development of targeted treatments and more precise management strategies.

## 1 Introduction

Irritable bowel syndrome (IBS) is a common condition, predominantly affecting young individuals, and is associated with a substantial burden in terms of impaired quality of life, reduced productivity, and increased healthcare utilisation (1). Despite its prevalence and impact, IBS remains a challenging condition for clinicians. Its pathogenesis is heterogeneous and incompletely understood, and treatment failure is frequent, with a considerable proportion of patients remaining refractory even to newer therapeutic options.

In the context of personalised medicine, stratification of patients according to underlying pathophysiological mechanisms is therefore warranted to optimise treatment outcomes. In this regard, the identification of biomarkers, potentially supported by artificial intelligence–based approaches, may play an important role.

In recent years, the identification of specific, predominantly clinical, features has allowed the recognition of several distinct subgroups within the IBS population, characterised by identifiable or presumed pathophysiological mechanisms. In one subset of patients, low-grade intestinal inflammation and immune activation appear to predominate, often in association with immune-mediated comorbidities such as coeliac disease or inflammatory bowel disease. In another subset, dysregulation of the gut–brain axis is prominent, involving alterations in gut microbiota, impaired motility, visceral hypersensitivity, and altered central nervous system processing (2, 3). A further group is characterised by brain health disturbances, including anxiety and depression, providing a rationale for the use of neuro-modulatory therapies.

### 2.1 The atopic endotype

More recently, growing attention has been directed towards an additional subgroup, often referred to as the “IBS atopy variant” (4–6). This phenotype is characterised by the presence of allergic comorbidities, most commonly allergic rhinitis and asthma, features of type 2 inflammation, and, in some patients, seasonal variability of gastrointestinal symptoms. Although the prevalence of this subgroup remains to be clearly defined, antiallergic therapies targeting mast cells and their mediators may represent a promising treatment strategy in selected patients (6).

Mast cells, key effector cells in allergic diseases, have long been implicated in IBS pathophysiology. Several mast cell abnormalities have been documented in IBS, including increased mast cell density, enhanced mediator release following stimulation, and close anatomical proximity to enteric nerve endings (2, 3). Hyperplasia and activation of mast cells, together with other immune cells such as eosinophils, have been linked to key features of IBS, including heightened sensitivity to luminal stimuli, altered motility and secretion, and increased intestinal permeability, as demonstrated in translational models (3).

Mast cell mediators, including histamine, proteases, prostaglandins, and leukotrienes, can augment the excitability of vagal and splanchnic afferent nerves, promote peripheral sensitisation, and stabilise nociceptor hyperexcitability. Mast cells also influence gastrointestinal motility by modulating smooth muscle contractility and myenteric plexus function. In addition, mast cell activation affects epithelial transport and barrier function; for example, histamine and tryptase contribute to chloride secretion via protease-activated receptor-2, potentially promoting diarrhoeal symptoms. The clinical relevance of mast cell-driven symptom generation is further supported by observations in mastocytosis, a clonal mast cell disorder, in which abdominal pain and diarrhoea are highly prevalent.

**Figure 1:**
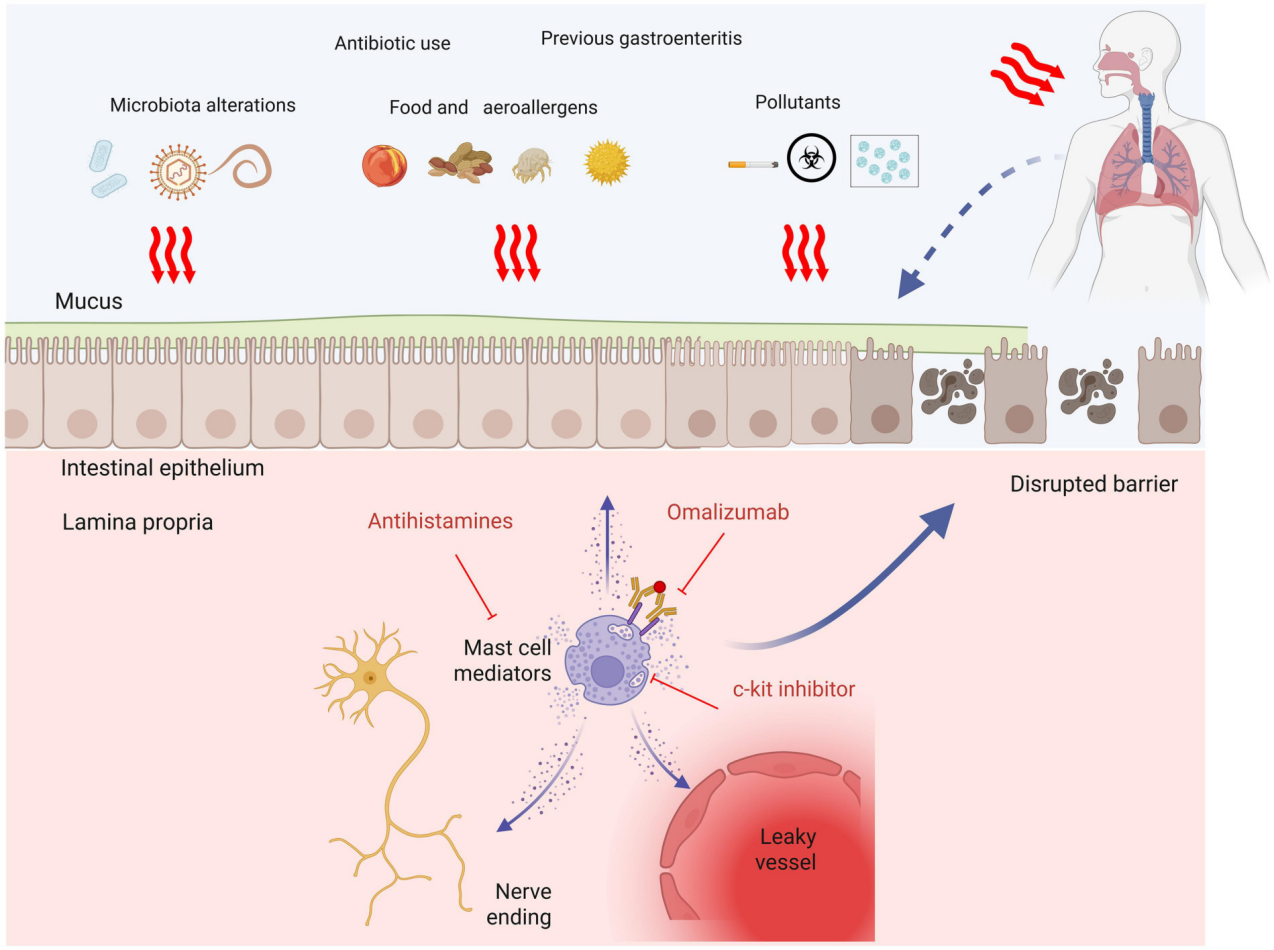
The figure depicts established and more recently characterized or proposed triggers associated with exacerbations of irritable bowel syndrome (IBS), particularly in its atopic variant. These triggers include previous gastrointestinal infections, antibiotic exposure, alterations in the gut microbiota, environmental pollutants, and possibly food- and respiratory allergens (such as pollens and house dust mite). Collectively, these factors contribute to epithelial inflammation and barrier disruption (red arrows), and subsequently to mast cell activation, through mechanisms that are only partly understood. These mechanisms may involve both IgE- and non-IgE–mediated pathways, potentially including direct mast cell activation. Activated mast cells release a wide spectrum of mediators, which stimulate nerve endings, induce smooth muscle contraction, and increase vascular permeability, thereby driving the occurrence of symptoms. In parallel, epithelial barrier dysfunction may arise, as illustrated in the right part of the panel, through epithelial cell apoptosis and detachment. Pharmacological strategies targeting mast cells are shown in red. These include inhibitors of mast cell activation (*e.g.*, anti-IgE monoclonal antibodies such as omalizumab), agents. Made with Biorender.

Several recent lines of evidence have strengthened the concept of an atopic endotype of IBS, providing a biological framework to explain symptom heterogeneity. Within this context, a particularly relevant research area is the identification of triggers that activate mast cells and influence IBS disease activity, with important diagnostic and therapeutic implications (Figure 1). Beyond microbial factors, stress, neurotransmitters, and food-related triggers, increasing attention has been directed towards allergenic stimuli. The potential mechanisms by which allergens may exacerbate IBS symptoms are summarised in Supplementary Box 1.

### 2.2 Aeroallergens and gut symptoms: emerging mechanisms and clinical perspective

While earlier studies focused mainly on food allergens as direct activators of intestinal mast cells, emerging evidence suggests that aeroallergens, both perennial and seasonal, may also contribute to symptom variability in IBS patients (7). Epidemiological data indicate a strong association between IBS and allergic diseases. In a Belgian study, IBS patients had a threefold higher risk of allergic rhinitis compared with controls, while patients with IBS experienced more severe rhinitis symptoms than individuals without IBS (6).

Seasonal variability of gastrointestinal symptoms provides further support for this association. In a recent monocentric study, patients with IBS-diarrhoea sensitised to grass pollen experienced a significant worsening of abdominal symptoms during the pollination season, whereas no seasonal variation was observed in patients sensitised to house dust mite, a perennial allergen (8). Importantly, patients receiving allergen-specific immunotherapy were excluded, and the use of systemic antiallergic therapies was limited, reducing potential treatment-related bias.

The mechanisms underlying aeroallergen-related gastrointestinal symptoms remain incompletely understood. While proteases from perennial allergens such as house dust mite may exert direct effects on the intestinal epithelium, the role of pollens appears more complex and may involve cross-reactivity with food allergens (9). It has also been hypothesised that small quantities of pollen allergens may reach the intestine intact and directly activate intestinal mast cells. However, convincing mechanistic evidence supporting this hypothesis is currently lacking.

Importantly, patients’ sensitisation profiles were not reported in these studies, and no explanation based on the physicochemical properties of the relevant allergens was provided. Only allergens that are resistant to heat, gastric acid, and proteolytic digestion are expected to reach the distal intestine in an intact, immunologically active form. Most pollen-related food allergens, such as Bet v 1 homologues, are heat- and acid-labile and are therefore degraded before reaching the intestine, typically inducing only local oral symptoms unless ingested under specific conditions, such as proton pump inhibitor use (10). In contrast, only a limited number of allergenic proteins, such as lipid transfer proteins, seed storage proteins, and oleosins, are sufficiently stable to elicit effector responses in the colon. However, sensitisation to these allergens is relatively uncommon in Central Europe and is usually associated with more severe food allergy phenotypes, making their involvement in IBS less likely (10).

An alternative explanation involves the translocation of inhalant allergens from the respiratory mucosa to the intestinal mucosa via a disrupted epithelial–vascular barrier. Increased vascular permeability, particularly in the context of active allergic rhinitis, may allow allergens to bypass the upper gastrointestinal tract and interact directly with gut mast cells. The association between IBS symptom severity and respiratory disease activity supports this hypothesis. Notably, vascular barrier disruption has recently been demonstrated in eosinophilic oesophagitis, a type 2 inflammatory disease characterised by allergic comorbidities, including allergic rhinitis (11).

## 3 Discussion

Beyond its pathogenetic relevance, assessment of mast cell activity and allergic sensitisation profiles in IBS may have diagnostic implications. Biomarkers reflecting mast cell activation, such as faecal tryptase, may help identify patients with a mast cell-driven phenotype, although their clinical utility remains under investigation.

From a therapeutic perspective, personalised strategies targeting mast cells and their mediators may be beneficial in patients with an atopic IBS phenotype. Mast cell stabilisers, antihistamines, and anti-IgE monoclonal antibodies have shown symptom improvement in IBS clinical trials (12). The Supplementary Table summarises the studies evaluating antiallergic medications in IBS. Other approaches aimed at disrupting mast cell–nerve interactions, such as tryptase inhibitors and neurokinin receptor antagonists, are currently under investigation. Combination therapies targeting both mast cell-related and neuro-modulatory pathways may represent a promising option for refractory cases.

An additional intriguing concept is whether modulation of mast cell-mediated inflammation at extraintestinal sites, such as the respiratory tract in allergic rhinitis or asthma, may improve gastrointestinal symptoms in IBS patients with a mast cell-driven phenotype. Optimised treatment of allergic rhinitis during pollination seasons might help prevent IBS exacerbations. Large, well-designed randomised controlled trials are needed to evaluate these strategies and to establish the diagnostic and predictive value of mast cell-related biomarkers.

Overall, the atopic endotype of IBS, characterised by allergic sensitisation and mast cell activation, may represent a treatable trait. Moreover, these findings are consistent with previous evidence highlighting the interplay between allergic and immune-mediated mechanisms in gastrointestinal disorders, further supporting the relevance of allergic pathways in functional conditions such as IBS (13). Nevertheless, despite increasing evidence supporting a role for allergic mechanisms in IBS, this phenotype is not currently considered in authoritative IBS classification frameworks (14). Future research priorities in this field are summarised in Supplementary Box 2.

In conclusion, identifying patients with a mast cell-driven phenotype and allergic sensitisation may enable clinicians, particularly in internal medicine settings, to implement mechanism-based, personalised interventions rather than relying solely on symptom-based management, thereby addressing key drivers of this complex syndrome.
