# Supplementary material for: Any role for respiratory allergens in IBS symptoms? From pathogenetic hypothesis to therapeutic implications
Source: Front Allergy. 2026 Jun 2;7:1844274. doi: 10.3389/falgy.2026.1844274 (PMC13269261; doi:10.3389/falgy.2026.1844274)
Supplement: Supplementary file 1 [file Supplementaryfile1.docx]

**Supplementary Box 1. Putative mechanisms of allergens as a trigger of IBS exacerbation**

- Direct IgE-mediated mast cell degranulation
- Non-IgE-mediated mast cell degranulation
- IgE-mediated mast cell degranulation through cross-reactivity between respiratory and food allergens
- Protease or enzymatic activity
- Microbiota alterations
- Epithelial and vascular barrier disruption

**Supplementary Box 2. Future research topics**

- Gut-specific and other biomarkers reflecting the atopic IBS subtype
- Experimental clinical trials assessing the role of anti-mast-cell mediator therapies in IBS with and without allergic sensitisation
- The role of different classes of allergens and routes of sensitisation (cutaneous, respiratory, gastrointestinal) in inducing IBS exacerbation
- Knowledge dissemination of the atopic subtype among clinical practitioners, including internists, gastroenterologists, allergists, nutritionists

**Supplementary Table. Studies evaluating antiallergic medications in IBS patients**

| **Therapeutic class** | **Drug** | **Clinical evidence** | **Main outcomes** | **Comments** | **Ref** |
| --- | --- | --- | --- | --- | --- |
| H1 antihistamines | Ebastine | RCT | Reduction in abdominal pain, improvement in global IBS symptoms and quality of life | All IBS variants were included | 15 |
| H2 antihistamines | Ranitidine, famotidine | CT | Symptom improvement | Trial duration: 6 months | 16 |
| Mast cell stabiliser | Disodium cromoglycate | Multicentre CT | Symptom improvement, especially in prick-test positive patients | Included IBS-D patients | 17 |
| Dual-action agents | Ketotifen | RCT | Increased threshold in patients with IBS with visceral hypersensitivity, reduced IBS symptoms and improved health-related quality of life | No effect observed in normosensitive IBS patients | 12 |
| Anti-IgE therapy | Omalizumab | Case series | Overall symptom improvement | Prescribed for concomitant chronic uritcaria | 18 |

Abbreviations. CT, clinical trial; IBS-D, irritable bowel syndrome, diarrhoea; RCT, randomised controlled trial.
